# Modelling survival

**DOI:** 10.7554/eLife.52959

**Published:** 2019-12-10

**Authors:** Teresa A Zimmers, Leonidas G Koniaris

**Affiliations:** 1 Department of SurgeryIndiana University School of MedicineIndianapolisUnited States; 2 Anatomy Cell Biology and PhysiologyIndiana University School of MedicineIndianapolisUnited States; 3 Biochemistry and Molecular BiologyIndiana University School of MedicineIndianapolisUnited States; 4 OtolaryngologyIndiana University School of MedicineIndianapolisUnited States; 5 Simon Cancer CenterIndiana UniversityIndianapolisUnited States; 6 Indiana Center for Musculoskeletal HealthIndiana UniversityIndianapolisUnited States; 7 Center for Cachexia Research, Innovation and TherapyIndiana University Purdue University IndianapolisIndianapolisUnited States

**Keywords:** sepsis, muscle, mitochondria, post-intensive care syndrome, chronic critical illness, Mouse

## Abstract

A new mouse model of sepsis can reproduce the long-term muscle weakness seen in patients who survive this life-threatening illness.

**Related research article** Owen AM, Patel SP, Smith JD, Balasuriya BK, Mori SF, Hawk GS, Stromberg AJ, Kuriyama N, Kaneki M, Rabchevsky AG, Butterfield TA, Esser KA, Peterson CA, Starr ME, Saito H. 2019. Chronic muscle weakness and mitochondrial dysfunction in the absence of sustained atrophy in a preclinical sepsis model. *eLife* **8**:e49920. doi: 10.7554/eLife.49920

Sepsis arises when the immune system produces an overwhelming response to an infection, which can lead to widespread blood clotting, shock (i.e., too little oxygen being delivered to tissues), multiple organ failure and, if left untreated, death. There are an estimated 31.5 million cases of sepsis worldwide per year (Fleischmann et al., 2016). Millions of children and newborns are affected by sepsis, and one-in-ten maternal deaths and half of all hospital deaths are due to this condition (Fleischmann-Struzek et al., 2018). The majority of hospital deaths occur in elderly patients suffering severe, chronic diseases such as cancer that predispose them to developing this life-threatening illness (Singer et al., 2019). It’s not only vulnerable people who are susceptible to sepsis, even healthy individuals suffering a new injury or infection can die from this condition. However, the number of patients dying from sepsis in the US and other economically developed nations is declining due to patients receiving earlier diagnosis and intensive care.

Although acute sepsis treatment has led to more survivors, it has become increasingly apparent that the life-threatening effects of sepsis do not end once a patient leaves the hospital (Fleischmann et al., 2016). In fact, patients have an elevated risk of mortality for at least two years following treatment (Prescott et al., 2016), and more than 20% of survivors will die within a year (Brakenridge et al., 2019). Even healthy individuals who survive sepsis rarely return to normal function (Prescott and Angus, 2018a; Prescott and Costa, 2018b). The underlying cause of this post-sepsis deterioration and late mortality is partly due to the body being weakened by the sepsis response, by the life-saving drugs administered to treat the sepsis, and by having reduced mobility when in intensive care. This leads to muscle wasting, as well as loss of muscle strength and performance. Eventually muscle mass recovers, but muscle strength does not, and this can leave a profound and lasting impact on survivors.

To understand how to treat these long-term effects researchers need an experimental model that can replicate these symptoms. Multiple rodent models are used to study the acute sepsis response by infecting their skin or tissue, by surgically puncturing their intestine, or by injecting bacterial toxins, bacteria or fecal preparations (Lewis et al., 2016). These models can be used to study how the body responds to infection and identify potential interventions that could reduce the rate of death in the short-term. However, there are very few models available for studying the later effects of sepsis. An ideal model would need to resemble an injury seen in patients; include interventions that mimic modern medical care, such as antibiotics and support for damaged organs; be able to clear the infection, recover and survive; and support long-term studies and functional analysis. Now, in eLife, Hiroshi Saito and co-workers at the University of Kentucky, Harvard Medical School and the University of Florida – including Allison Owen as first author – report a mouse model that meets all of these requirements (Owen et al., 2019; Figure 1).

**Figure 1.**
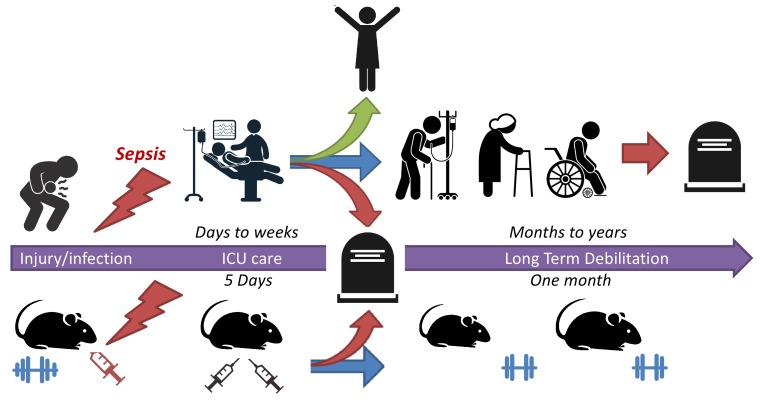
A mouse model for studying sepsis survival. Sepsis is a life-threatening illness that occurs when the immune system overreacts to an injury or infection. Patients with early diagnosis of sepsis who are treated in an intensive care unit (ICU) have a much greater chance of survival (green arrow). However, a significant number of survivors suffer from long-term debilitating effects (blue arrow) and late mortality (red arrow). To better understand how to treat these long-term effects, Owen et al. have developed a mouse model that replicates the treatment outcomes of patients who had survived sepsis: 16-month-old mice were given an injury that induced sepsis and then given the same fluids and drugs patients would receive when in the ICU. These mice exhibited the long-term muscle weakness that commonly occurs in sepsis survivors.

Image credit: Icons are copyright of Greyjj/Dreamstime.com.

Unlike typical mouse studies that use young or adolescent mice, Owen et al. used 16-month-old mice which are the equivalent of middle-aged humans (age 50 years). To mimic the disease process seen in intestinal conditions (such as severe appendicitis), a fecal bacterial suspension was injected into the mice’s abdominal cavity. These bacteria were rapidly absorbed into the body and the mice quickly developed sepsis with blood-borne bacteria. After a 12 hour period of illness without intervention (to mimic worsening disease), the mice were given fluids and antibiotics to simulate the care received by patients when in intensive care. This rescue protocol treated the sepsis, shock, and organ injury over the next five days and improved the survival rate of mice from 0–14% to 75%.

The mice that survived sepsis demonstrated a large and persistent loss of strength. Both male and female mice lost 30–35% of their muscle strength two weeks after becoming infected with sepsis, even when accounting for loss in muscle size. This weakness lasted a month, even once muscle mass had returned and all evidence of infection and inflammation had disappeared. Owen et al. found that this weakness was not the result of low food intake, but due to damage to mitochondria in the mice’s skeletal muscle cells.

The main role of mitochondria is to provide cells with energy, so it is possible that the damaged mitochondria cannot produce enough energy for muscle contraction, and their faulty metabolism may even perpetuate further cell damage. One could speculate that therapies that protect mitochondria during anti-sepsis treatment, or drugs that re-habilitate damaged mitochondria, could prevent or reduce the functional decline in sepsis survivors.

This model of sepsis survival should stimulate new research not only into acquired weakness, but also into the cognitive, emotional and physical impairments sepsis survivors face following intensive care (Inoue et al., 2019). Careful molecular and cellular studies using this mouse model could identify new therapeutic targets and interventions that improve the long-term outcomes of sepsis survivors.
